# Glass Lightweight Aggregates from Glass Cullet and Mining and Food Industry Carbonate Waste

**DOI:** 10.3390/ma15031223

**Published:** 2022-02-06

**Authors:** Isabel Padilla, Aurora López-Delgado, Maximina Romero

**Affiliations:** “Eduardo Torroja” Institute for Construction Sciences, IETcc–CSIC, 28033 Madrid, Spain; isabel.padilla@ietcc.csic.es (I.P.); alopezdelgado@ietcc.csic.es (A.L.-D.)

**Keywords:** glass lightweight aggregates, LWA, glass cullet, carbonated waste, eggshell, mussel shell, magnesite waste, food industry waste, thermal shock, linear expansion

## Abstract

Lightweight aggregates are extensively used in construction and other industrial applications due to their technological characteristics. The extraction of natural aggregates results in serious environmental effects. Thus, within the circular economy concept, the valorization of waste through the optimization of materials and product design is encouraged. In this work, glass lightweight aggregates were prepared from mixtures of white glass cullet and carbonate wastes from mining (wastes originating from the extraction, manufacture and marketing of magnesite and its derivatives) and the food industry (eggshell and mussel shell). The effects of different processing parameters, such as the particle size of the base glass, percentage of the blowing additive, shaping method, heating rate, temperature and processing time, were evaluated. The results indicate that the mineralogical composition of the blowing agent and the particle size of the base glass are the two processing parameters with the greatest impact on expansion efficiency. Thus, glass artificial aggregates were obtained with characteristics similar to those of commercial products (density values ranged between 0.3 and 0.8 g/cm^3^ and mechanical strength between 0.7 and 1.5 MPa) from thermal shock expansion treatments in the temperature range 800–900 °C and with dwell times no longer than 15 min.

## 1. Introduction

Aggregates are crucial materials in the manufacture of concrete and mortar, prefabricated products (blocks, beams, pavements, etc.), road construction (wearing courses, asphalt agglomerates, bases and subbases), railway ballast, breakwaters (ports, dikes and dams), foundations and fillings (bridges, airports, streets, tunnels, buildings and water pipelines). The aggregate sector is by far the largest of the non–energy extractive industries, and the use of aggregates in the construction industry represents 88% of the total consumption. Although this industry has traditionally used dense aggregates, there is currently a growing demand for lightweight aggregates (LWAs), which, due to their structural and technological characteristics, are increasingly used in the manufacture of concrete, lightweight prefabricated products and insulating barriers [1].

LWAs are aggregates with a particle density not exceeding 2000 kg/m^3^ or a bulk density not exceeding 1200 kg/m^3^. These values derive from a porous structure with a uniform pore distribution; closed cells; and a hard, densely sintered outer surface. In addition to the construction sector, LWAs are used in considerable quantities in numerous industrial applications (lime and plaster manufacturing, basic metallurgy, the glass industry, the basic chemical industry, etc.).

The extraction of natural aggregates inevitably affects the environment, as most of the process is carried out in opencast quarries or gravel pits. These exploitations result in serious environmental alterations, as they gradually transform the landscape and cause the degradation of natural resources. According to the latest UEPG data (Union Européenne des Producteurs de Granulats, European Aggregates Association) [2], the European demand for aggregates is 3 billion ton per year. Of this, 86% comes from natural resources (47% from crushed rock and 39% from sand and gravel), and only 14% comes from marine, artificial, recycled and in situ reused aggregates. UEPG and the Fédération Internationale du Recyclage (FIR) have jointly created the European Platform for Recycled Aggregates (EPRA) to promote the use of recycled aggregates. The EPRA argues that the construction industry should be committed to promoting sustainable development, minimizing potential adverse effects on the environment and encouraging appropriate resource management.

However, concern about the impacts of human activity on the natural environment has led to the signing of three landmark agreements in recent years: the Paris Agreement on climate change, the 2030 Agenda for Sustainable Development and the United Nations Environment Assembly Ministerial Declaration “Towards a pollution–free planet”. Similarly, the term circular economy has been adopted, which is a model of production and consumption in which the value of products, materials and resources is retained in the economy for as long as possible, waste generation is minimized, and waste that cannot be minimized is used as much as possible. A circular economy looks, among others, at the valorization of waste through the optimized design of materials, products and systems [3].

In view of this situation, the search for new ways of manufacturing artificial lightweight aggregates is a matter of priority. Thus, this paper shows the results of a study conducted to produce glass lightweight aggregates from compositions that only incorporate waste as raw materials. As such, glass packaging waste is considered a base glass, and different carbonate wastes from the food industry (eggshell and mussel shell) and mining (waste originating from the extraction, manufacture and marketing of magnesite and its derivatives) are considered foaming agents.

In 2020, EU–28 glass production reached a volume of 35.9 million ton [4]. The worldwide production is approximately 195 million annually, of which 46% is container glass [5]. According to EUROSTAT, in 2018, the member states of the European Union produced 18.7 million ton of container glass waste [6]: 26% was recycled, 13% was destined for combustion with energy recovery, and the remaining 61% ended up in a landfill. Regarding eggs, the worldwide production was 90 million ton in 2019 [7]. Considering that the shell represents approximately 10% of the egg by weight [8], the annual production of eggshell waste can be estimated to be 9 million tons. For mussel shell waste, more than 14 × 10^6^ ton of bivalves are produced each year in aquaculture, of which 2 × 10^6^ ton correspond to mussel production [9]. Considering that the mussel shell represents approximately 75% by weight, the annual production of mussel shell waste can be evaluated at 1.5 × 10^6^ ton. Although some studies have focused on the use of these food wastes in different applications, such as catalysts for biodiesel production [10,11,12], papermaking [13,14], the removal of heavy metals and soluble microbial products from wastewater [15,16] or the synthesis of construction materials [17], hydroxyapatite [18,19] or active carbon [20], the current situation is that most eggshell and mussel shell waste originating worldwide is destined for landfills. Therefore, based on the concept of a circular economy, research on new ways of valorization for this type of waste is of great importance, and the manufacture of glass lightweight aggregates could be a suitable alternative.

In recent decades, the literature has reported several studies on the incorporation of waste in the manufacture of foamed glass, which is produced from a mixture of crushed or granulated glass and an additive that promotes foaming. This mixture is heated to a temperature at which the glass becomes a viscous liquid, and in turn, the additive decomposes and releases gases, which, unable to migrate to reach the surface, are trapped within the glass mass, thereby creating closed cells. As base glass, the use of waste glass of different natures has been reported, such as fluorescent tubes [21], cathode ray tubes [22,23,24,25,26,27], bottles [28,29,30], flat glass [31], float glass [27], calcosodic glass [26,32,33] and glass cullet [34,35]. For foaming additives, the use of several materials, such as alkaline or alkaline earth carbonates of both mineral origin [36,37,38] and animal origin, such as eggshell [21,22,29,39] or porcine bone [40]; NaOH [27]; SiC [31,35,41]; metallic oxides; graphite [41,42,43]; organic compounds, such as glycerin, gelatin, starch or saccharose [30]; and agro–food wastes, such as banana leaves [28] or tobacco residue [44], has been reported. However, it should be noted that the main objective of the aforementioned studies is the production of glass foams to be applied as a thermal or acoustic insulator. Nevertheless, other types of materials can be obtained from waste glass, such as glass lightweight aggregates, which may have applications in different sectors, such as in the manufacture of lightweight mortars and concretes; as lightweight backfill; in the construction of retaining walls or bridge piers and abutments; or as drainage material in sports facilities, parks and gardens.

The manufacture of glass lightweight aggregates is not sufficiently explored in the literature and needs to be deeply investigated. Therefore, the novelty of this study is the sustainable manufacture of glass lightweight aggregates. Moreover, to the authors’ knowledge, the use of wastes originating from the extraction of magnesite has not been previously studied for this purpose. The optimal parameters for the production of glass LWAs from mixtures of white glass cullet with carbonates of mineral (magnesite production residues) and food industry waste (eggshells and mussel shells) origins are established, and the effect of composition and experimental parameters on the technological characteristics of the LWAs obtained is determined.

## 2. Materials and Methods

The base glass used for the synthesis of glass lightweight aggregates (LWAs) was a white glass cullet (GC) supplied by VERALLIA S. A (Zaragoza, Spain), a glass container manufacturer. Three different wastes from the extraction, manufacture and marketing of magnesite and its derivatives were used as mineral foaming additives. These wastes, namely, carbonate F (CF), flotation tailing (FT) and carbonate PC8 (PC8), were supplied by Magnesitas de Navarra (Zubiri, Spain). Moreover, eggshell (ES) and mussel shell (MS), both wastes from the food industry, were also used as foaming agents.

Prior to use, the glass cullet required a conditioning step consisting of grinding in an oscillating vibratory disk mill TS 100 model (SIEBTECHNIK TEMA, Madrid, Spain), sieving to a particle size below 1 mm and homogenization in a mixer/mill SPEX 8000 (SPEX Sample Prep, Rickmansworth, UK). The FT residue was subjected to oven drying at 120 °C for at least 24 h, grinding in a planetary mill PM 100 (RETSCH, Haan, Germany) and sieving to a particle size below 1 mm. The ES and MS residues were oven dried at 120 °C for at least 24 h, mechanically fragmented, ground in a blade mill and sieved to a particle size below 297 µm. CF and PC8 additives did not require conditioning and were used as received.

The raw materials were characterized by different analytical techniques. Chemical composition was determined by X–ray fluorescence (XRF) in a wavelength–dispersive spectrometer (S8 Tiger model, Bruker, Champs–sur–Marne, France). Pressed pellets of 10 g raw material without additives were prepared for the measurements. Loss on ignition (LOI) was calculated by heating the samples at 1000 °C for 1 h in a Pt crucible. The amorphous or crystalline nature of the raw materials was determined by X–ray diffraction (XRD) (D8 Advance model, Bruker, Champs–sur–Marne, France) using CuKα radiation. The diffractograms were recorded in the interval 2θ = 2–65° with a scan rate of 0.07 s per step. XRD data processing was performed with the EVA Diffrac Plus 13.0 program, and crystalline phase identification was performed with the Powder Diffraction Data file (PDF) database. The thermal behavior of the raw materials was evaluated by differential thermal and thermogravimetric analysis (DTA/TG) (Setaram Labsys) using platinum crucibles, a controlled atmosphere (synthetic airflow) and calcined alumina as reference material.

To study the ability of the different carbonate–based wastes to yield foaming, the effect of different processing parameters was evaluated, as summarized below:

- Base glass particle size: <63 µm, 63–100 µm, 100–250 µm, 250–500 µm and <1 mm.
- Percentage of foaming agent: 2.5, 5, 7.5, 10 and 15% by weight.
- Type of shaping: The shaping of specimens by pressing and hand shaping was evaluated. For the preparation of pressed pellets, cylindrical specimens (2 cm diameter and approximately 1.5 cm in height) were formed from 2 g of a homogenized mixture of raw materials (glass cullet and foaming additive) slightly moistened (3% water). The shaping was performed by uniaxial pressing at 3 MPa for 30 s in a semiautomatic hydraulic press (Mignon–S model Nannetti S.r.l., Faenza, Italy). For hand shaping, 30 g of raw material mixtures was prepared and mixed with water (23% by weight). Spherical specimens (~1 cm in diameter and ~0.75 g in weight) were formed from each mixture. Prior to the foaming process, the specimens were dried at 105 °C for 24 h.
- Heating rate: 10, 20, 30, 40 and 50 °C/min. In addition, the heating effect was evaluated by thermal shock, which consisted of introducing the samples into an oven preheated to the blowing temperature.
- Firing temperature: 700, 800, 900 and 1000 °C.
- Foaming time: 5, 10, 15, 20, 30 and 45 min.

To evaluate the impact of each processing variable, the remaining parameters were set as shown in Table 1.

After the manufacture, the specimens were characterized by determining the linear expansion (LE) experienced during the heat treatment and by measuring the bulk density (BD) and compressive strength (σ_c_) of the foamed specimens. The above characteristics were determined in 10 samples, and the mean values of the measurements are presented. Linear expansion, expressed as a percentage, was determined according to Equation (1):

LE = (Ø_g_/Ø_e_ ×100) − 100, (1)

where Ø_g_ and Ø_e_ are the diameters of the dry green specimen and those after the foaming process, respectively.

Bulk density was determined by a direct method based on the weight and dimensions (by measuring with a digital caliper with ±0.01 mm error) of specimens.

The compressive strength was determined according to standard EN 658–2:2002 [45] in a 1000 N SERVOSIS testing machine. The tests were carried out by applying the load by means of compression plates connected to the load cell and on the mobile crosshead and using a strain rate that allowed the specimens to fail in less than 1 min.

## 3. Results and Discussion

### 3.1. Waste Characterization

**Figure 1:**
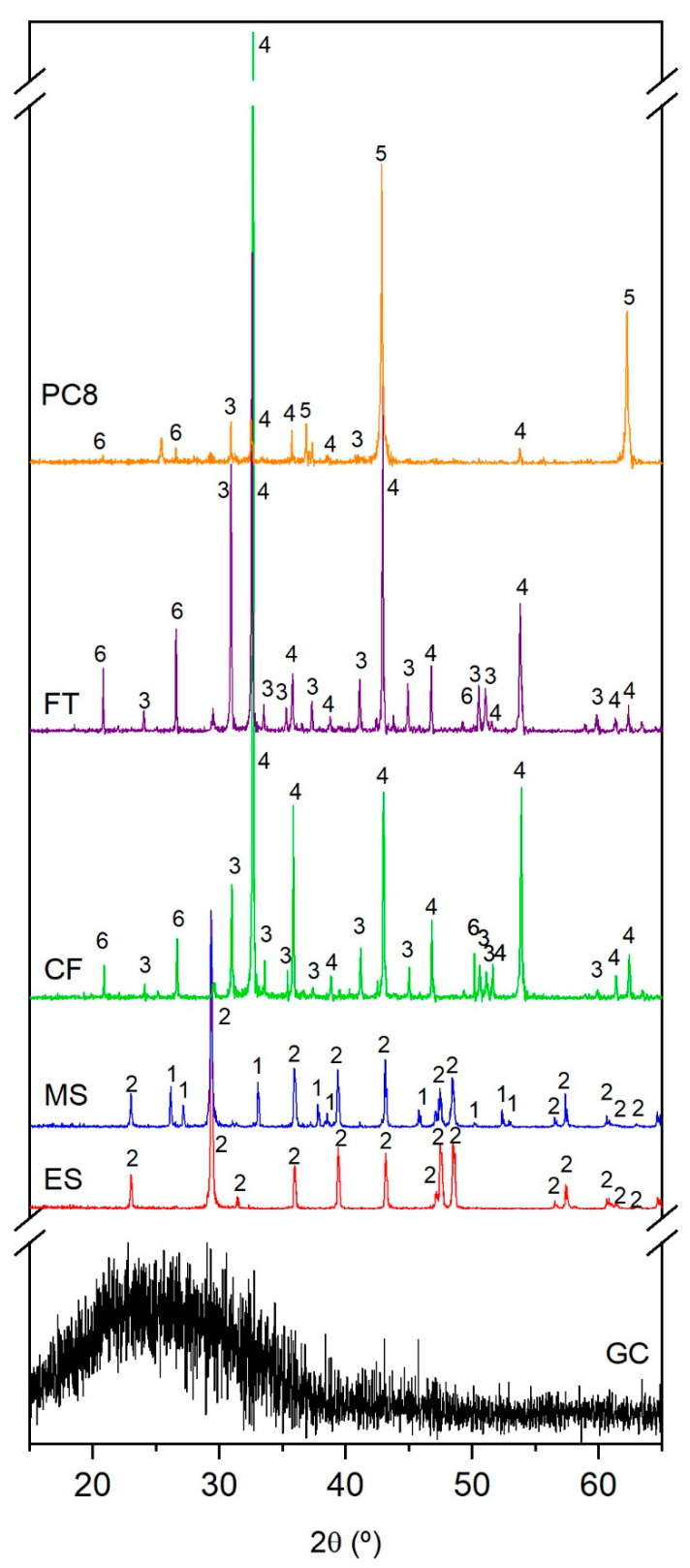
XRD patterns of raw materials: glass cullet (GC), eggshell (ES), mussel shell (MS), carbonate F (CF), flotation tailing (FT) and carbonate PC8 (PC8). (1: aragonite (CaCO_3_), 2: calcite (CaCO_3_), 3: dolomite (CaMg(CO_3_)_2_), 4: magnesite (MgCO_3_), 5: periclase (MgO) and 6: quartz (SiO_2_)).

**Figure 2:**
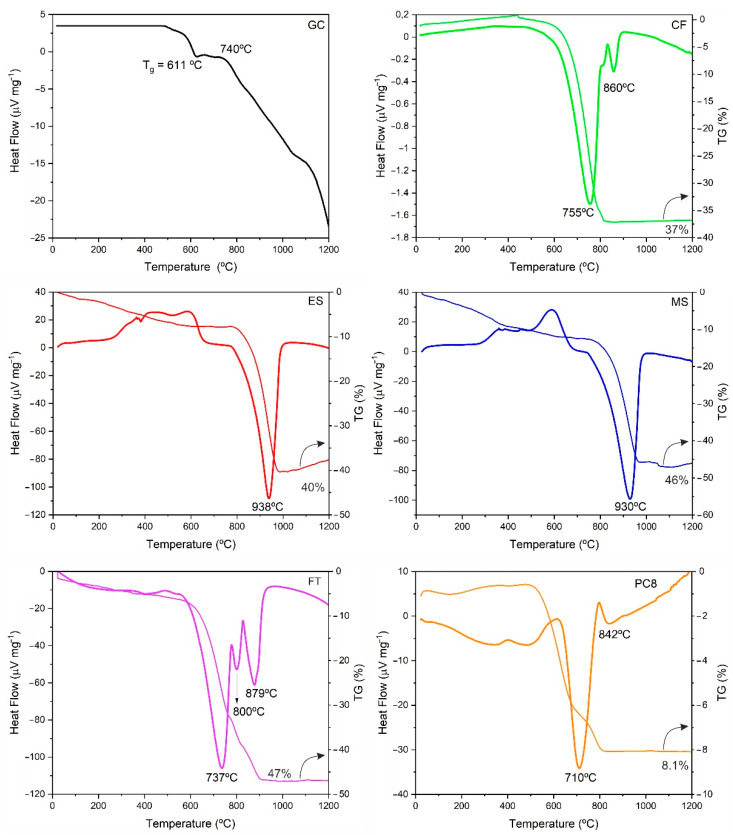
DTA/TG curves of raw materials: glass cullet (GC), eggshell (ES), mussel shell (MS), carbonate F (CF), flotation tailing (FT) and carbonate PC8 (PC8).

The chemical composition of the raw materials used as the base glass and foaming agents is shown in Table 2, and the corresponding XRD patterns and DTA/TG curves are shown in Figure 1 and Figure 2, respectively.

The glass cullet presents a typical soda–lime glass composition, formulated mainly from silicon oxide (vitrifying agent), sodium carbonate (flux) and calcium carbonate (stabilizer). It is a completely amorphous material, as indicated by its XRD pattern (Figure 1), in which only a diffuse halo, indicative of the absence of long–range atomic order, is observed in the interval 2θ = 10–40°.

Regarding the thermal behavior, the DTA curve of the glass cullet shows a glass transition temperature, T_g_, at approximately 610 °C (Figure 2). The density, hardness and rigidity decrease from T_g_, and the glass is turned into a viscous plastic state that is suitable for trapping gases from the thermal decomposition of the foaming additives, which is suitable for the development of LWAs [39].

The carbonate residues of mineral origin (CF, FT and PC8) are composed of mixtures of magnesite (MgCO_3_), dolomite (CaMg(CO_3_)_2_) and quartz (SiO_2_) according to their DRX patterns in Figure 1. Their DTA curves (Figure 2) show, from 500 °C onwards, different endothermic peaks due to the decomposition of the carbonate crystalline phases. Thus, the intense peaks centered at approximately 750 and 870 °C correspond to the decomposition of dolomite in two stages: first, the partial decarbonation of CaMg(CO_3_)_2_ to form MgO and CaCO_3_ and then the decomposition of the latter [22]. A lower intensity peak centered at 800 °C corresponds to the decarbonation of the magnesite present in the initial samples [47,48]. The chemical composition of these raw materials is consistent with their mineralogical composition, with magnesium, calcium and silicon oxides as major components and alumina and iron oxide as minor constituents.

The carbonate residues from the food industry (ES and MS) are composed mainly of calcium carbonate (CaCO_3_) (Figure 1). The X–ray diffractogram of the eggshell residue indicates the presence of calcite as the only crystalline phase, while the mussel shell waste is composed of a mixture of the polymorphs calcite and aragonite [46,49]. Their DTA curves show an endothermic drop from 700 to 750 °C, leading to a peak centered at approximately 935 °C, which corresponds to the decarbonation of CaCO_3_ (Figure 2). Prior to the decomposition of calcium carbonate, these residual materials show a weight loss of approximately 10% due to the decomposition of the organic matter. For their chemical composition, as expected, both wastes are composed of calcium oxide as the main constituent.

### 3.2. Pressing–Formed Pellets

**Figure 3:**
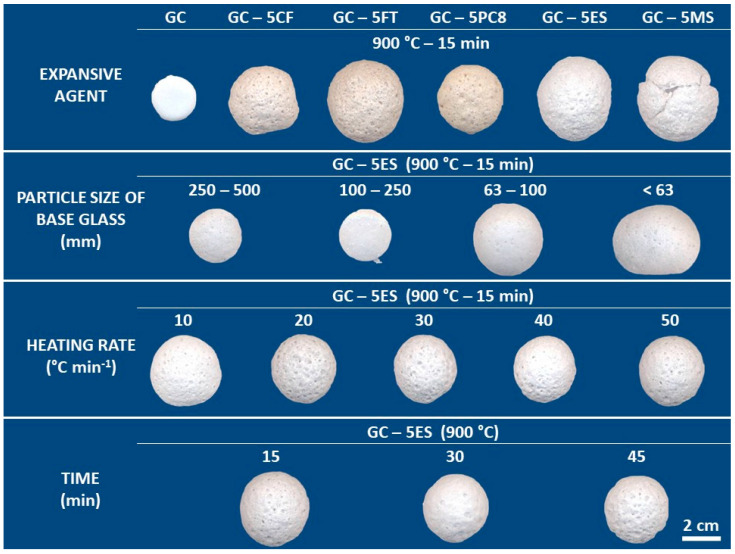
Appearance of the glass lightweight aggregates after the expansion tests performed with different processing parameters on pressing pellets.

Figure 3 shows the appearance of the materials prepared after the expansion tests with different processing parameters (blowing agent, particle size of the base glass, heating rate and dwell time). The evaluated processing parameters have a different effect on the degree of foaming, as described below.

**Figure 4:**
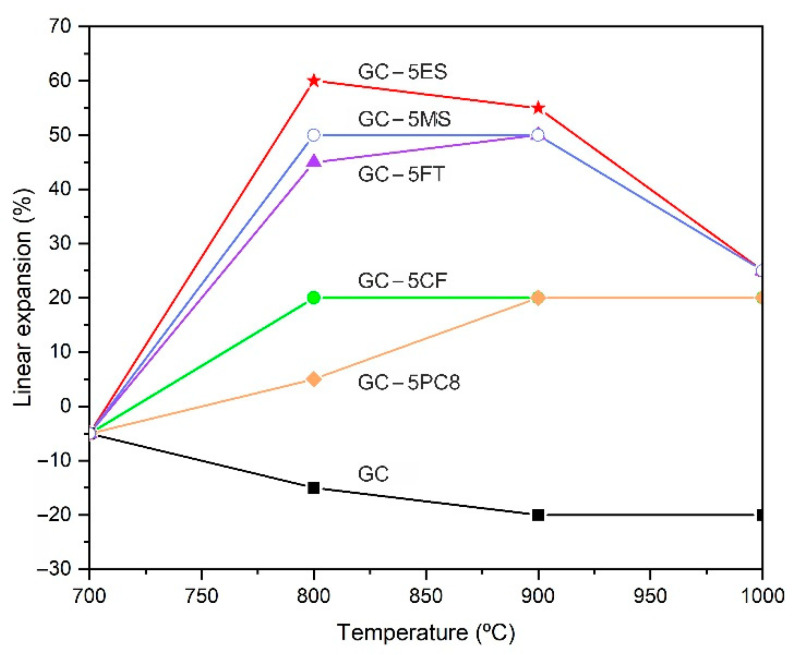
Evolution of linear expansion (LE) with temperature in pressed pellets formulated with different additives (5 wt. %) and foamed for 15 min (base glass particle size < 1 mm).

Figure 4 shows the evolution of the linear expansion (LE) with temperature in the pressed specimens formulated with different additives (5% by weight) and heat treated for 15 min. As a reference, Figure 4 also shows the curve corresponding to the glass cullet without the addition of a blowing agent. As expected, the glass cullet undergoes shrinkage (approximately 20%) with an increase in temperature because of a sintering process in which the open porosity decreases while the glass particles are rearranged by a viscous flow mechanism. In the samples with an added blowing agent, the sintering of the glass particles starts in an analogous way. At 700 °C, all the samples experience a linear shrinkage of approximately 5%, but the progress of the sintering process is slowed down by the decomposition of the additives and the consequent release of CO_2_ gas, which promotes the expansion of the specimens. Figure 4 indicates that all the blowing agents studied are effective in inducing expansion in the manufacture of LWAs, with the ES, MS and FT residues being the most effective. With these additives, a maximum linear expansion, LE_max_ = 45–60%, is achieved in the temperature range 800–900 °C. At higher temperatures, the sintering strength predominates over the expansion strength, and as a result, the linear expansion decreases in all cases to a value of approximately 25%. However, the CF and PC8 additives result in a lower degree of linear expansion (LE_max_ = 20%), and the sintering and expansion forces are balanced so that once the maximum expansion is reached, it is maintained with an increasing temperature.

The difference in behavior observed in the expansion capacity of the different wastes used as additives is related to the starting temperature of carbonate decomposition [39,44]. In the CF and PC8 additives, the decarbonation reactions of MgCO_3_ (the beginning of the endothermic drop in Figure 2) start at 520 and 600 °C, respectively, and develop up to 800 °C. In both cases, the decarbonation onset temperatures are lower than the T_g_ of the base glass (611 °C), so the gases released at the beginning of the decarbonation reaction can easily escape between the open porosity between the glass particles. In the case of the ES and MS additives, which give rise to the highest expansion values, the onset of the CaCO_3_ decarbonation reactions (780 and 750 °C, respectively) takes place at temperatures well above the T_g_ value. In this case, the released gases find a more sintered matrix in which the open porosity has been considerably reduced, becoming trapped in the matrix and leading to its expansion. In the case of the FT additive, the decomposition of MgCO_3_ starts at 570 °C, similar to the CF and PC8 additives. However, the presence of CaCO_3_ in this residue promotes the release of gases at temperatures above 800 °C, and, therefore, a degree of expansion comparable to that achieved with the ES and MS additives is reached. In view of these results, a composition containing 5% by weight of the ES additive was selected to study the effect of the particle size of the base glass and the heating rate on the expansion.

**Figure 5:**
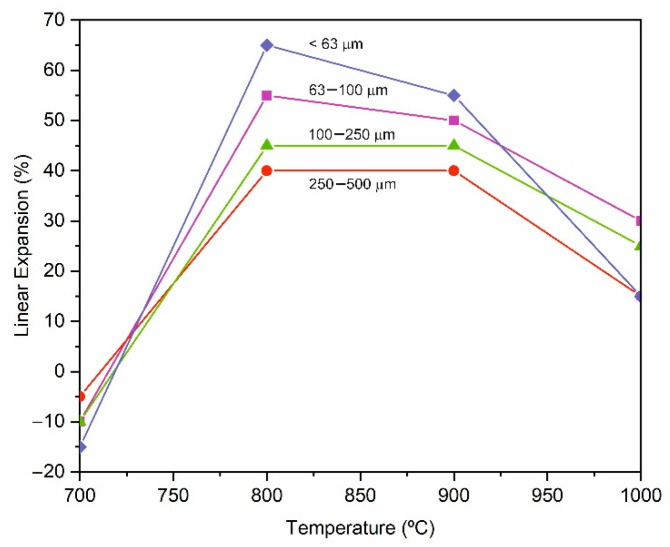
Evolution of linear expansion (LE) with temperature in pressed pellets of the composition GC–5ES formulated from different–particle–size glass cullet fractions and heat treated for 15 min.

Figure 5 shows the evolution of the linear expansion with temperature as a function of the particle size of the base glass for the pressed specimens with 5 wt. % eggshell (GC–5ES). At 700 °C, all specimens experience volume shrinkage, which is greater the smaller the initial glass particle size. Amorós et al. [50] stated that under the same pressure conditions, a higher content of smaller particles leads to green specimens of lower porosity, which, however, contain higher pores. Therefore, as the glass particle size decreases, green compaction increases, and, consequently, the pressed specimens contain less open porosity between particles, and the early stages of sintering are favored, thus achieving higher specimen shrinkage. At 800 °C, when thermal decomposition of the additives occurs, the gases released have fewer open porosity paths to escape to the surface of the specimens, becoming trapped inside the specimens and leading to greater expansion.

The maximum expansion values are obtained in all cases in the temperature range 800–900 °C, with mean values of LE_max_ = 40% for the fraction with the largest particle size (250–500 µm) and LE_max_ = 60% for the pressed pellets prepared from the fraction <63 µm. The smaller the base glass particle size, the higher the expansion of the specimens. This effect of the particle size on the expansion of the LWAs is similar to that reported by König et al. [51]. When comparing Figure 4 and Figure 5, it is observed that the CV–5ES composition prepared from a fraction with a particle size <1 mm (Figure 4) reaches maximum expansion values similar to those obtained with the fraction <63 µm (Figure 5). This result indicates a very hard milling of the base glass, so the <1 mm sample is composed mainly of particles <63 µm in size. Consequently, the rest of the study was carried out with the <1 mm sample.

**Figure 6:**
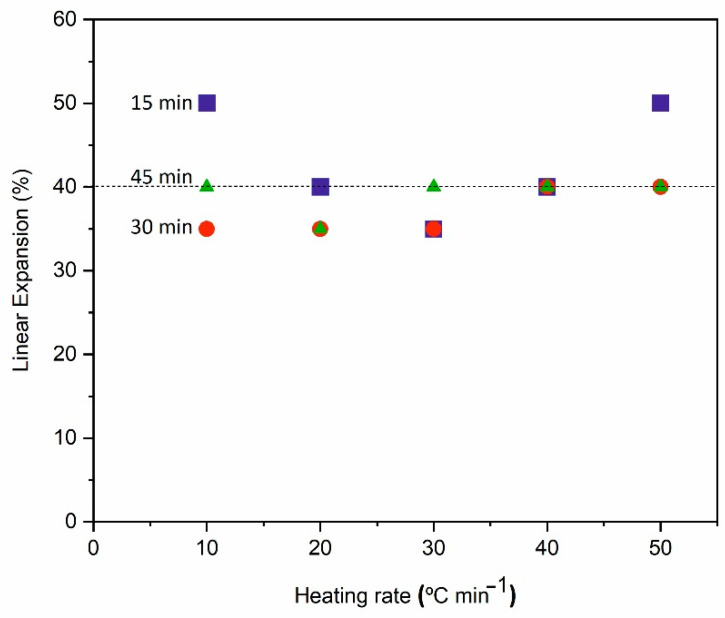
Evolution of linear expansion (LE) with heating rate in pressed pellets of GC–5ES thermally treated at 900 °C for 15 min (base glass particle size <1 mm).

In the previous tests, the pellets were foamed by thermal shock; i.e., the green pellets were introduced into the preheated oven at the treatment temperature. To evaluate the effect of the heating rate, different foaming tests were carried out in which the green pellets were introduced into the oven at room temperature and subjected to a heating ramp up to 900 °C. Figure 6 shows the evolution of the linear expansion with heating rate and treatment time in the pressed specimens formulated with 5% by weight of ES (GC–5ES) and heat treated for 15 min. None of the processing parameters evaluated (heating rate and dwell time) are observed to have a significant effect on the expansion value of the specimens, reaching an average expansion value of 40%, slightly lower than the value of 55% reached in the foaming tests by thermal shock and a 15 min dwell time.

### 3.3. Hand–Formed Pellets

**Figure 7:**
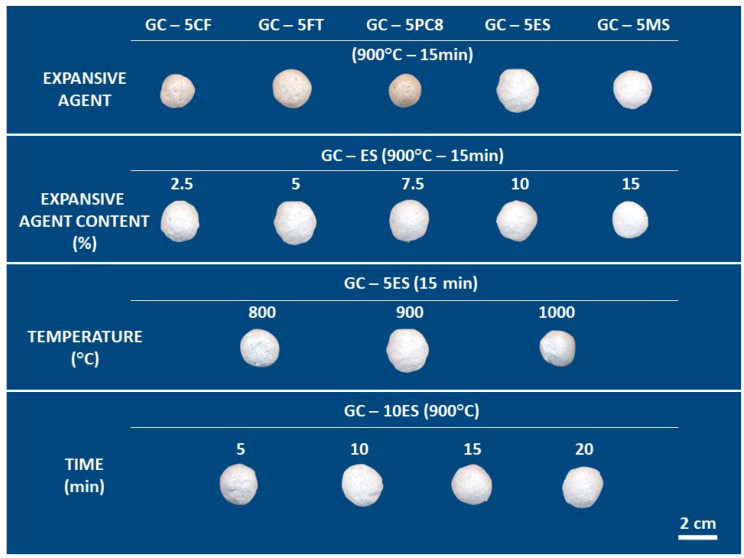
Appearance of the glass lightweight aggregates after the expansion tests performed with different processing parameters on hand–formed pellets.

The results described below were obtained on spherical pellets (~1 cm in diameter) shaped by hand. The appearance of the LWAs obtained after expansion tests with different processing parameters (type and percentage of blowing agent, temperature and expansion time) can be seen in Figure 7. The results indicate that the molding method (manual or mechanical pressing) does not influence the expansion process, since no significant differences were observed in the linear expansion values with respect to those obtained with pressed specimens (Figure 3).

**Figure 8:**
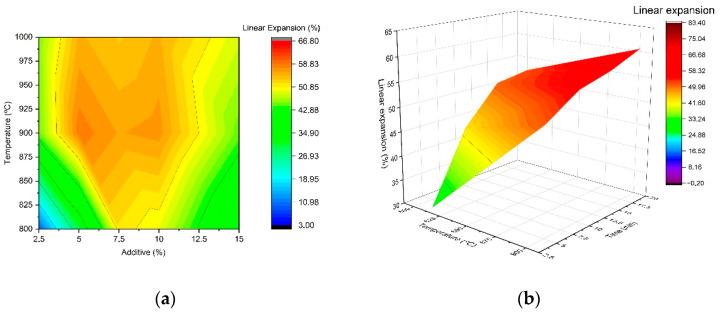
Evolution of linear expansion (LE) of hand–formed pellets with (**a**) temperature and expansive agent percentage, and (**b**) temperature and dwell time.

Figure 8 shows the evolution of the linear expansion of these pellets as a function of the percentage of foaming agent added, the temperature and the treatment time. The different compositions, prepared with the addition of the different foaming additives, are represented in the same plot, as all of them follow a similar trend. Thus, regardless of the additive used, Figure 8a shows that, in the spherical specimens, there is a wide expansion range, with the maximum value being obtained at a temperature of 900 °C. In addition, expansion values higher than 50% are achieved for foaming agent percentages between 5 and 10 wt. %. Therefore, to evaluate the effect of expansion time, compositions with 10 wt. % addition of blowing agent were selected, and the green bodies were expanded at temperatures in the range of 800–900 °C for different treatment times. Figure 8b presents the evolution of linear expansion with temperature and treatment time. The expansion rate is very high, and treatment times as short as 2.5 min result in an expansion of approximately 30%. The degree of expansion increases with treatment time, although after 15 min, the variation observed in the expansion values is not significant.

However, for the selection of the processing parameters, not only the expansion experienced by the green bodies but also the properties of the obtained glass lightweight aggregates in terms of density and mechanical strength must be considered. Similar to the observations in the study of pressed pellets, the different additives used produce LWAs with different degrees of expansion and, therefore, different density values. Thus, food industry wastes (ES and MS) induce a greater expansion and give rise to aggregates with a lower density. However, the PC8 residue is the additive that promotes the lowest degree of expansion and, accordingly, the highest density values. As mentioned above, the different behavior of the additives is related to the different value of the starting temperature of the decomposition of the carbonates used in relation to the T_g_ of the base glass. The profile of the compressive strength variation curves is quite similar to that of the density curves, indicating that both properties are related.

**Figure 9:**
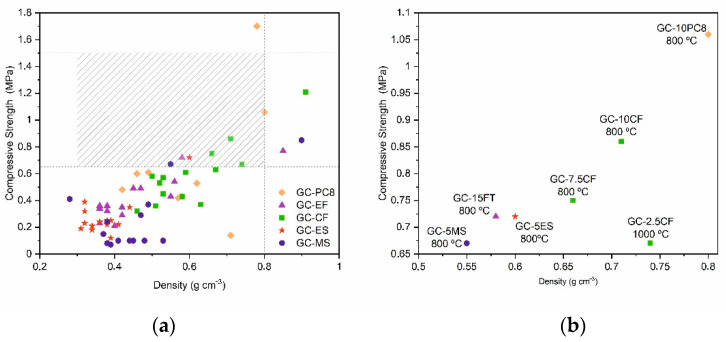
(**a**) Location of all the compositions studied in terms of their density and compressive strength values, and (**b**) magnification of the region dashed in (a), which corresponds to the typical range of values showed by commercial LWAs.

Figure 9 shows the location of all the compositions studied in terms of their density and compressive strength values. The dashed lines delimit the range of density (0.3–0.8 g/cm^3^) and mechanical strength (0.65–1.5 MPa), which corresponds to commercial lightweight aggregates with similar grain sizes. In general, the glass aggregates obtained in this study present density values in the range of commercial lightweight aggregates (Figure 9a), although their compressive strength values are mostly below the minimum value of 0.7 MPa. Nevertheless, in the magnification of Figure 9b (shaded region), several compositions of the LWAs meet both the density and the mechanical strength requirements, *viz*.: GC–5MS, GC–5ES, GC–7.5CF, GC–10CF, GC–10PC8 and GC–15FT prepared at 800 °C and GC–2.5CF at 1000 °C.

The results indicate that all the expansive additives investigated in this study result in glass lightweight aggregates, with 800 °C being the most suitable processing temperature for the materials obtained to have the appropriate density and strength properties for application as LWAs.

Finally, it is not possible to establish, in a general way, the optimal percentage of the expansive agent to be added, since the expansion capacity will depend on its nature and its physical–chemical characteristics. In fact, the results highlight that the crystallographic and morphological characteristics of calcium carbonate have a great influence on the consistency and mechanical strength of glass lightweight aggregates. Thus, the lowest values of both properties were obtained when mussel shell was used as an additive. This result can be attributed to the presence of aragonite, a calcite polymorph with prismatic crystals that can hinder the intimate link between the particles in the initial pressed pellets [46]. Most of the compositions (2.5–10%) prepared with CF waste from mining produced LWAs with excellent properties. Moreover, the waste that allows the major percentage incorporation is FT waste from mining, and LWAs can be obtained by adding up to 15 wt. % of this waste.

## 4. Conclusions

Glass lightweight aggregates were prepared from mixtures of white glass cullet and carbonate wastes from mining and the food industry. Based on the findings from the study of the effects of different processing parameters, the following conclusions can be drawn:

- The expansion capacity of the different residues used as additives depends on the relative position of the onset decomposition temperature of the carbonates with respect to the glass transition temperature of the base glass.
- All the studied blowing agents are effective in promoting the expansion of the specimens, with eggshell, mussel shell and flotation tailings being the most effective.
- The heating rate does not produce a significant effect on the expansion values. Thermal shock heating is shown to be the most effective procedure for obtaining glass lightweight aggregates.
- The mineralogical composition of the blowing agent and the particle size of the base glass are the two processing parameters that have the greatest influence on the degree of expansion.
- All the foaming additives investigated in this study give rise to glass lightweight aggregates, with 800 °C being the most suitable processing temperature for the materials obtained to have the appropriate density and strength properties for their application as lightweight aggregates.

The use of mining and food waste along with packaging glass cullet in the manufacture of glass lightweight aggregates could make an important contribution to the circular economy and environmental sustainability.

## 5. Patents

“Procedure to obtain glass lightweight aggregates” Spanish Patent Application P202130406, 6 May 2021.

## Figures and Tables

**Table 1:** Processing parameters used in the different foaming tests.

	Fixed Parameters					
Variable Parameter	Shaping	Glass Particle Size	Foaming Agent (wt. %)	Heating Rate (°C/min)	Foaming Temperature (°C)	Foaming Time (min)
Foaming temp	Pressing	<1 mm	5	Thermal shock	Variable	15
Glass particle size	Pressing	Variable	5	Thermal shock	700, 800, 900, 1000	15
Heating rate	Pressing	<1 mm	5	Variable	900	15
Foaming time	Pressing	<1 mm	5	10, 20, 30, 40, 50	900	Variable
Foaming temp	Handly	<63 µm	2.5, 5, 7.5, 10, 15	Thermal shock	Variable	15
Foaming agent percentage	Handly	<63 µm	Variable	Thermal shock	800, 900, 1000	15
Foaming agent	Handly	<63 µm	10 (*)	Thermal shock	800, 850, 900	Variable

(*) Different additives were introduced at 10 wt. %, except MS (5 and 7.5 wt. %).

**Table 2:** Main chemical composition (expressed as wt. %) of the raw materials, determined by XRF.

Oxide	Glass Cullet (GC)	Carbonate F (CF)	Flotation Tailing (FT)	Carbonate PC8 (PC8)	Eggshell (ES) *	Mussel Shell (MS) *
SiO_2_	72.17	14.70	9.52	3.63	0.13	0.19
Al_2_O_3_	1.84	3.41	1.18	0.83	-	-
CaO	15.04	17.44	32.27	11.85	97.24	97.68
MgO	0.30	61.93	54.96	61.40	0.40	0.10
Na_2_O	9.81	1.62	1.49	3.10	-	0.30
K_2_O	0.66	0.90	0.59	1.74	0.67	0.70
SO_3_	0.17	-	-	10.08	0.98	0.45
Fe_2_O_3_	0.00	7.11	5.48	5.28	-	-
Cl	-	-	-	1.07	0.27	0.28
SrO	-	-	-	-	-	0.20
P_2_O_5_	-	-	-	0.61	0.21	-
MnO	-	-	-	0.15	-	-
V_2_O_5_	-	-	-	0.14	-	-
LOI	2.02	50.67	49.59	12.89	47.00	46.10

* [46].

## Data Availability

Not applicable.
